# Bezafibrate for the treatment of dyslipidemia in patients with coronary artery disease: 20-year mortality follow-up of the BIP randomized control trial

**DOI:** 10.1186/s12933-016-0332-6

**Published:** 2016-01-22

**Authors:** Yaron Arbel, Robert Klempfner, Aharon Erez, Ilan Goldenberg, Sagit Benzekry, Nir Shlomo, Enrique Z. Fisman, Alexander Tenenbaum, for the BIP Study Group

**Affiliations:** Department of Cardiology, Tel Aviv Medical Center, Affiliated to the Sackler Faculty of Medicine, Tel Aviv University, Tel Aviv, Israel; Leviev Heart Center, Sheba Medical Center, Tel Aviv, Affiliated to the Sackler Faculty of Medicine, Tel Aviv University, Tel Aviv, Israel; Cardiovascular Diabetology Research Foundation, Holon, Israel; The Israeli Association for Cardiovascular Trials, Tel Hashomer, Israel; Heart Research Follow-up Program, University of Rochester, Rochester, NY USA

**Keywords:** Bezafibrate, Triglycerides, Coronary disease, Prognosis, Mortality, Lipids, Cholesterol, HDL

## Abstract

**Background:**

Recent data support the renewed interest in hypertriglyceridemia as a possible important therapeutic target for cardiovascular risk reduction. This study was designed to address the question of all-cause mortality during extended follow-up of the BIP trial in patients stratified by baseline triglyceride levels.

**Methods:**

In the BIP trial 3090 patients with proven coronary artery disease were randomized to bezafibrate 400 mg/day or placebo. All-cause mortality data after 20 years of follow-up, were obtained from the National Israeli Population Registry. Patients with hypertriglyceridemia (triglycerides ≥200 mg/dL, n = 458) were equally distributed among the study groups (15 % in both placebo and bezafibrate groups).

**Results:**

During follow-up 1869 patients died (952 in placebo vs. 917 in bezafibrate group). Following multivariate adjustment allocation to bezafibrate was associated with small but significant 10 % mortality risk reduction (HR 0.90; 95 % CI 0.82–0.98, p = 0.026). Variables associated with significantly increased mortality risk were history of a past MI, NYHA class, diabetes, age, higher BMI and glucose level. In patients with hypertriglyceridemia multivariate analysis demonstrated a 25 % all-cause mortality risk reduction associated with allocation to bezafibrate (HR 0.75, CI 95 % 0.60–0.94; p = 0.012). In patients without hypertriglyceridemia bezafibrate had no significant effect on long-term mortality.

**Conclusions:**

During long-term follow-up bezafibrate-allocated patients experienced a modest but significant 10 % reduction in the adjusted risk of mortality. This effect of bezafibrate was more prominent among patients with baseline hypertriglyceridemia (25 % mortality risk reduction).

**Electronic supplementary material:**

The online version of this article (doi:10.1186/s12933-016-0332-6) contains supplementary material, which is available to authorized users.

## Background

There is a growing body of recently published genetic and epidemiological evidences which demonstrated a causal role of triglycerides (TG) and TG-rich lipoproteins in the pathogenesis of atherosclerosis and particularly coronary artery disease (CAD) [1–5]. These data support the renewed interest in hypertriglyceridemia as a possible important therapeutic target for cardiovascular risk reduction [6–8]. Historically, one of the main aims of the Bezafibrate Infarction Prevention (BIP) trial was to assess the effect of reducing TG levels (alongside with rising of HDL-C levels) on cardiac risk in patients with established CAD [9]. During the course of the study, TG levels were reduced by 21 % and HDL-C increased by 18 % among patients that had received bezafibrate. Nevertheless, despite these favorable lipid-modifying effects, bezafibrate therapy was associated with only a non-significant trend of a reduction of the incidence of primary end point (fatal or non-fatal myocardial infarction or sudden death). However, a significant 39.5 % reduction in the primary end point in patients with high baseline TG (≥ 200 mg/dl) was observed. Also during post hoc analysis of the BIP trial we have shown that bezafibrate significantly reduced the incidence of myocardial infarction in patients with the metabolic syndrome [10]. The relatively short mean follow-up of 6.2 year which could be achieved during the double-blind phase of the BIP trial precluded a determination of whether bezafibrate affected total mortality. We hypothesized that early favorable effects of bezafibrate on lipids and myocardial infarction during the original trial period could be translated in a subsequent reduction in total mortality during a longer-term observation. Therefore, this study was designed to address the question of mortality during extended 20-year follow-up of the BIP trial.

## Methods

### The BIP trial

The BIP trial evaluated the effect of bezafibrate versus placebo on major coronary events and mortality in CHD patients. Details of the study design have been previously published [9, 11–13]. Briefly, 3090 male and female patients 45–74 years of age with a history of MI and/or stable angina pectoris and a lipid profile of serum total cholesterol between 180 and 250 mg/dL, low-density lipoprotein cholesterol (LDL-C) ≤180 mg/dL (≤160 mg/dL for patients <50 years), HDL-C ≤45 mg/dl, and triglycerides ≤300 mg/dL were randomized to bezafibrate 400 mg/day or placebo between May 1990 and January 1993 and followed up over a mean period of 6.2 years (median 6.2 years; interquartile range [IQR] 5.3–6.8 years).

Stable angina pectoris confirmed by coronary angiography, and/or radio- nuclear studies or standard exercise tests. The main exclusion criteria were insulin-dependent diabetes mellitus, severe heart failure, unstable angina pectoris, hepatic or renal failure, known sensitivity to bezafibrate, or current use of lipid-modifying drugs.

After discontinuation of the study medication, patients were observed for coronary events for an additional period, bringing the total follow-up time to a mean of 8.2 years (median 7.9 years; interquartile range 7.2–8.7 years). This study reports all-cause mortality data obtained in 2014 from the National Israeli Population Registry presenting 20 years follow-up [IQR 12–22.6]. The identification number recorded during enrollment was matched to mortality data stored at the national register and verified by matching date of birth. The current analysis is based on over 52,100 patient-years data.

### Study population

In the present analysis we included all the subjects included in the original BIP randomized study with the exception of 19 subjects that did not take any study medication. Excluded patients were equally distributed between the Bezafibrate and placebo arm (9 and 10 patients, respectively). IRB approval was obtained both for the original study and for the current long term follow-up data acquisition.

### Statistical analysis

Variables were expressed as mean ± SD, and categorical data were summarized as percentages. Baseline characteristics of patients with patients with TG ≥200 mg/dL by the original treatment allocation group were compared using the Chi-square test for categorical parameters and Student *t* test or Mann–Whitney for continuous variables, as appropriate. Similarly, we compared the pre-specified subgroups of subjects with baseline triglycerides levels ≥200 mg/dL to those with lower levels of triglycerides. The supplemental online data includes characteristics and comparison of the entire study population by original allocation to bezafibrate or placebo.

Survival curves were constructed by the Kaplan–Meier method, and the significance of the variation between them was assessed using a Log-rank test. We compared survival for the entire study population by original treatment allocation (placebo vs. bezafibrate), followed by survival analysis by treatment allocation in the pre-specified sub-groups of subject with TG ≥200 mg/dL and <200 mg/dL.

We used multivariate Cox proportional hazards regression modeling in order to explore the adjusted risk reduction associated with bezafibrate treatment in the entire study population and separately in the subgroup of patients with TG ≥200 mg/dL. Covariates were selected if univariate association was significant (p < 0.05) or clinical studies have demonstrated robust association with prognosis in previous studies. The following covariates were selected using the best subset method: age, gender, BMI, prior MI, history of diabetes mellitus, reported hypertension, NYHA functional class ≥2, and baseline serum values of HDL and glucose. An additional multivariate model was constructed including lipid-lowering medication initiated during the BIP study or the extended follow-up period (total 8 years’ duration) introduced as a time dependent covariate. Proportionality of hazard assumption was verified in all models.

We further undertook interaction term analysis in order to explore mortality associated with fibrate vs. placebo treatment by TG group (below 200 mg/dL vs. ≥200 mg/dL) with the covariates described above.

All statistical tests were two-sided, and a P value of less than 0.05 was considered to indicate statistical significance. The P values for interaction are reported. Analyses were carried out with the use of SPSS software, version 22 (IBM Inc.) and SAS, version 9.3.

## Results

The present study included patients from the original BIP cohort: 1548 patients allocated to the bezafibrate group and 1542 patients allocated to the placebo group. Clinical characteristics, laboratory values, and medical therapy were similar in the 2 original study treatment groups (Additional file 1: Table A). Nearly 80 % of the patients had a history of MI, and 10 % had treated diabetes mellitus. Beta-blockers were prescribed to nearly 40 % of study patients, calcium-channel blockers to 50 %, and angiotensin-converting enzyme inhibitors to 12 %.

Patients with triglycerides ≥200 mg/dL (n = 458) were equally distributed among the 2 study groups (15 % in both placebo and bezafibrate groups). There was no significant difference in characteristics of patients with TG ≥200 mg/dL randomized to the placebo (n = 224) vs. fibrate (n = 234) treatment arm (Table 1).

**Table 1:** Baseline Clinical and Laboratory characteristics of patients with baseline TG ≥200 mg/dl by original study allocation

	Bezafibrate group (n = 234)	Placebo group (n = 224)	P value
Clinical characteristics			
Age, years	58 ± 7	58 ± 7	0.62
Male	210 (90)	199 (89)	0.75
Hypertension	78 (33)	80 (36)	0.61
DM	28 (12)	21 (9)	0.37
BMI, kg/m^2^	28 ± 4	28 ± 3	0.81
NYHA functional class ≥2	71 (30)	53 (25)	0.41
AP class ≥2	72 (30)	53 (23)	0.40
Prior MI	173 (74)	171 (76)	0.60
COPD	4 (2)	8 (4)	0.22
Medical therapy			
Anti-platelets	157 (67)	155 (69)	0.63
Beta-blockers	116 (49)	94 (42)	0.10
Nitrates	114 (51)	127 (54)	0.47
Ca^2+^-blockers	107 (46)	116 (52)	0.11
ACE inhibitors	30 (13)	27 (12)	0.80
Diuretics	27 (11)	36 (16)	0.16
Non-study LLD	136 (58)	122 (54)	0.40
Laboratory values			
Glucose	104 ± 18	103 ± 19	0.76
Total cholesterol	215 ± 18	216 ± 18	0.46
HDL-C	31 ± 5	32 ± 5	0.14
LDL-C	138 ± 17	140 ± 17	0.74
Triglycerides	235 ± 25	235 ± 29	0.94
Fibrinogen	361 ± 74	355 ± 75	0.37

Values are presented as n (%) or mean ± SD

*AP* angina pectoris, *ACE* angiotensin-converting enzyme, *BMI* body mass index, *COPD* chronic obstructive pulmonary disease, *DM* diabetes mellitus, *HDL-C* high-density lipoprotein cholesterol, *LDL-C* low-density lipoprotein cholesterol, *LLD* lipid-lowering drug, *MI* myocardial infarction, *NYHA* New York Heart Association

Compared to patients with TG <200 mg/dL patients with baseline TG ≥200 mg/dL presented with similar clinical features, with the exception of a higher BMI and younger age. Laboratory values differed significantly, including lower HDL and lower LDL and higher fasting glucose levels (all p < 0.001; Additional file 2: Table B).

### Entire study population mortality

**Fig. 1:**
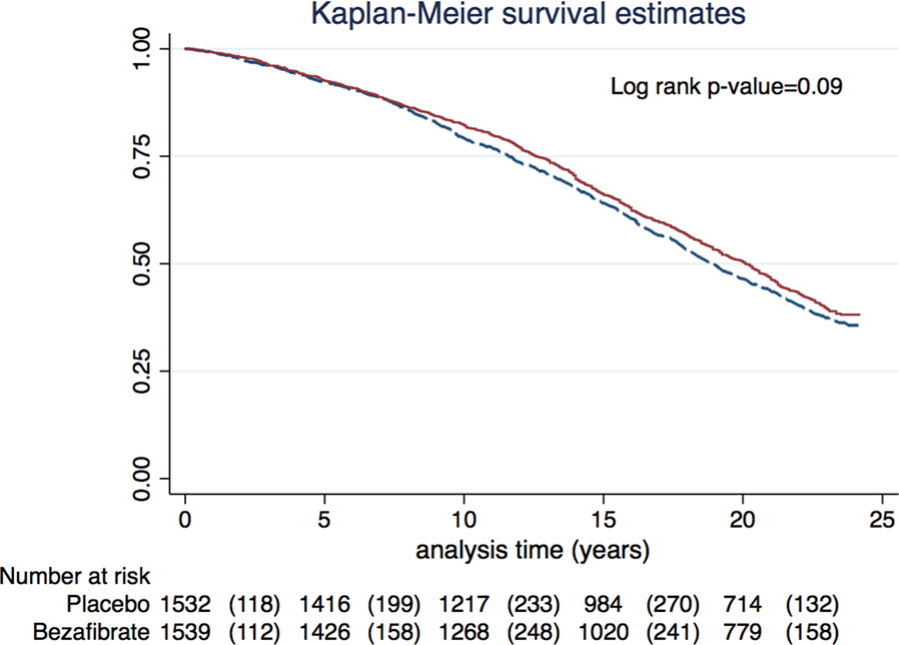
Long-term survival estimates by study drug allocation for the entire study population

During the follow-up period 1869 patients died (61 %), 952 (62 %) in the placebo group and 917 (60 %) in the bezafibrate group (Fig. 1). Following multivariate adjustment, allocation to the bezafibrate arm was associated with significant 10 % mortality risk reduction (HR 0.90; 95 % CI 0.82–0.98, p = 0.026; Table 2). Variables associated with significantly increased mortality risk were history of a past MI (HR 1.45; CI 0.92–0.98), NYHA class >1 (HR 1.13; CI 1.04–1.22), diabetes (HR 1.34; CI 1.14–1.58), older age, higher BMI and glucose levels (all p < 0.01; Table 2).

**Table 2:** Independent predictors of all-cause mortality at 20 years of the entire study cohort

	HR	95 % CI		P value
		Lower	Upper	
Fibrate treatment	0.90	0.82	0.98	0.026
Past MI	1.45	1.30	1.64	<0.001
Glucose (per 1 mg/dl)	1.01	1.005	1.01	<0.001
Age (per year increment)	1.09	1.08	1.10	<0.001
BMI (per unit)	1.03	1.01	1.04	<0.001
NYHA functional class ≥ II	1.13	1.04	1.22	0.005
TG ≥200 mg/dl	1.04	0.92	1.18	0.52
Diabetes mellitus	1.34	1.14	1.58	<0.001

Model further adjusted for hypertension, gender, baseline HDL and use of non-study lipid lowering medication as a time dependent covariate

### Mortality in TG sub-groups

Next, we divided our cohort according to their baseline triglyceride levels by original study allocation. Patients with baseline triglycerides ≥200 mg/dL allocated to placebo (n = 224) and bezafibrate groups (n = 234) were compared. Patients that were treated with bezafibrate had a non-significant unadjusted survival benefit (57 vs. 62 %; Log rank p value = 0.14).

**Fig. 2:**
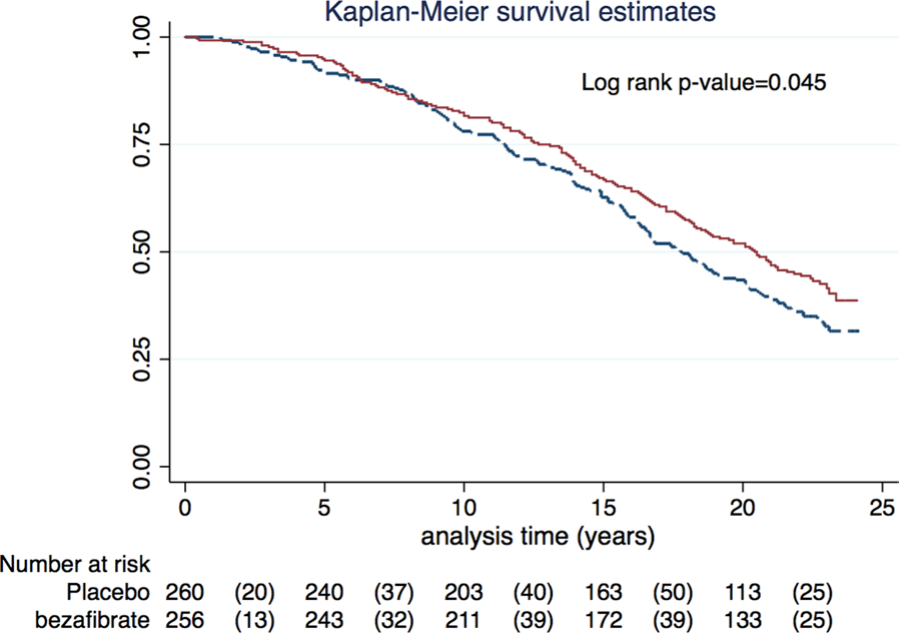
Adjusted survival probability by original study allocation of bezafibrate vs. placebo in patients with TG ≥200 mg/dL^&^. ^&^ Cox proportional hazard regression model adjusted for age, gender, hypertension, past MI, baseline glucose, BMI, NYHA ≥ II, HDL-C and diabetes status

Multivariate analysis demonstrated a 25 % adjusted all-cause mortality risk reduction in patients with hypertriglyceridemia treated by bezafibrate (HR 0.75; CI 95 % 0.60–0.94; p = 0.012; Fig. 2). Variables associated with significantly increased mortality risk in this subgroup were: age, past MI, use of non-study lipid lowering medication, and diabetes mellitus (Table 3).

**Table 3:** Independent predictors of all-cause mortality at 20 years in the sub group with baseline TG ≥200 mg/dL

	HR	95 % CI		P value
		Lower	Upper	
Fibrate treatment	0.75	0.60	0.94	0.012
Past MI	1.53	1.15	2.00	0.004
Glucose	1.00	0.99	1.01	0.12
Age (per year increment)	1.08	1.07	1.11	<0.001
BMI (per unit)	1.01	0.98	1.05	0.39
NYHA functional class ≥ II	1.09	0.89	1.33	0.41
HDL-C (per 1 mg/dl)	1.00	0.97	1.02	0.76
Diabetes mellitus	1.88	1.36	2.61	<0.001

Model further adjusted for hypertension, gender and non-study lipid lowering medication as a time dependent covariate (all p > 0.05)

**Fig. 3:**
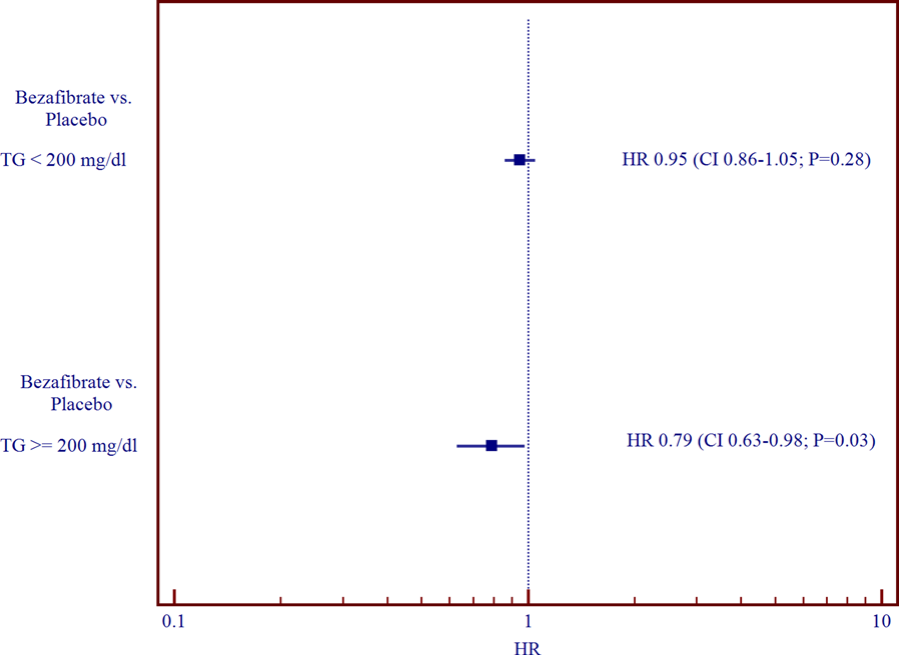
Independent effect of the adjusted risk reduction associated with bezafibrate vs. placebo treatment by pre-specified triglyceride group interaction*. P value for interaction of TG group by treatment allocation = 0.11. *Model further adjusted for age, gender, HDL-C, diabetes, past MI, BMI and glucose levels

Interaction term analysis demonstrated that the adjusted risk reduction associated with fibrate treatment compared to placebo is associated with a 21 % risk reduction (HR 0.79; CI 0.63–0.98; p = 0.03) in subjects with elevated TG whereas this effect was not significant in the group with lower TG values (p for interaction = 0.11; Fig. 3).

## Discussion

There are two main findings in this long-term study which based on the intention-to-treat principle with more than 52,000 person-years of follow-up. First, patients treated during original BIP trial period by bezafibrate experienced a modest but significant 10 % reduction in the adjusted risk of mortality compared with patients allocated to the original placebo group. Second, the long-term benefit of bezafibrate was more prominent among patients with hypertriglyceridemia (25 % mortality adjusted risk reduction).

Fibrates are used in clinical practice for the past half century mainly due to their ability to decrease TG. All fibrates are peroxisome proliferators-activated receptors α agonists with ability to enhance the oxidation of fatty acids in liver and muscle and reduce the rate of hepatic lipogenesis, thereby reducing secretion of very-low-density lipoprotein TG. Other important effects of fibrates include an increase of HDL level, activity of lipoprotein lipase, the size of LDL particles and a decrease in the apolipoprotein CIII concentration [14, 15].

The beneficial effects of all major fibrates (gemfibrozil, fenofibrate, bezafibrate) on cardiovascular events could be clearly demonstrated only in patients with dyslipidemia (mainly hypertriglyceridemia) or metabolic syndrome. Metabolic syndrome is an important prognostic sign [16]. In patients without these metabolic abnormalities these effects were absent [9, 17–21]. In a meta-analysis of dyslipidemic subgroups from 5 randomized control trials (RCT) with fibrates totaling 4726 patients, a 35 % relative risk reduction in cardiovascular events was observed compared with a non-significant 6 % reduction in patients without dyslipidemia [22]. Meta-analysis performed in a general population [23] reflecting a blend of effects in patients with and without dyslipidemia (45,058 participants) effect of fibrate therapy was reduced, producing only a modest but still significant 10 % RR reduction for major cardiovascular events and a 13 % RR reduction for coronary events. In these circumstances, the main determinant of the overall results of the fibrate’s trial is mainly dependent of the number of the included appropriate patients with dyslipidemia and/or metabolic syndrome.

From the modern point of view, patients who fulfilled inclusion criteria of the BIP trial should been treated primary by statins and not by fibrates. In fact, about 50 % of the patients in the BIP trial were presented with high LDL-C and low TG and probably did not need fibrates at all. In 1994 the Scandinavian Simvastatin Survival Study (4S) conclusions became available [24] and statins were administered to an increasing number of patients participating in the BIP trial in direct discordance with its protocol. Because of worse lipids profile of placebo-allocated patients they received statins in significantly greater proportion during the late period of the trial. Consequently, the wide use of non-study statins led to attenuation of the bezafibrate’s effect. After the cessation of the BIP trial the rate of use of statins increased substantially in entire study population and eventually was similar in both treatment groups [25].

After publication of the results from the extended phase of the UKPDS trial [26] the concept of “glycemic legacy” was widely discussed. In the 10-year post-trial follow-up, patients with type 2 diabetes originally allocated to intensive hypoglycemic treatment had a significant reduction in the risk of all-cause mortality after the cessation of randomized interventions. These results were obtained although there were no longer differences in HbA1c values between patients originally assigned to conventional or intensive-treatment groups.

To the best of our knowledge, our study is the first which demonstrated in patients with CAD and particularly in patients with hypertriglyceridemia the presence of long term “metabolic memory” for lipids-modified effects of bezafibrate. Our data together with the results from the extended phase of the UKPDS and STENO 2 trials [26–28] provide evidence that the beneficial results of the effective management of cardio-metabolic risk factors could be seen over many years even after the cessation of treatment. Pooled together, these findings support the possible positive role of a “metabolic legacy” for patients with high cardiovascular risk, rather than only a “glycemic legacy” for diabetic patients.

Currently statins are the cornerstone of the treatment and prevention of cardiovascular diseases related to atherosclerosis including CAD [29–32]. Nonetheless, despite the almost universal use of statins in the setting of secondary prevention of CAD, significant residual cardiovascular risk is still present, especially in patients with hypertriglyceridemia and metabolic syndrome [1, 33]. The current guidelines recommend considering combination fibrate-statin therapy for patients when statin therapy alone is not adequate to achieve lipid goals [29, 32, 34]. Despite the strong theoretical background, there are only few hard outcomes data regarding combined bezafibrate and statin treatment: one small randomized study [35] and number of observational studies [36–38]. In these observations bezafibrate/statin treatment was a safe and was associated with a lower incidence of major cardiovascular events compared with statins alone. Beneficial effects of long-term combination therapy with bezafibrate and ezetimibe in patients with dyslipidemia were also reported [39]. Probably, combined bezafibrate/statin or bezafibrate/ezetimibe therapy will be more effective in achieving a comprehensive lipid control and residual cardiovascular risk reduction and theoretically might prevent statin-associated new-onset diabetes [40].

### Study limitations

The present study is limited by the post hoc nature of the analysis of the BIP study. It should also be noted that the significant long-term effects of bezafibrate therapy could be observed only after adjustment for important established prognostic predictors including initiation of statins during the double-blind phase of the BIP trial. We do not have information regarding medications or events after the cessation of the BIP trial beyond all-cause mortality nor can we account for changes in medical practice and management guidelines over the long follow-up period. Therefore, further studies are needed to determine the possible long-term mortality benefit of bezafibrate in the current era of universal statin-based secondary prevention in CAD patients.

## Conclusions

Patients treated during the original BIP trial period by bezafibrate experienced a modest but significant 10 % reduction in the adjusted risk of mortality during extended follow-up of 20 years. The long-term benefit of bezafibrate was more prominent among patients with baseline hypertriglyceridemia (25 % mortality risk reduction). The present findings suggest that bezafibrate in patients with CAD and hypertriglyceridemia could be associated with a significant long-term mortality reduction that extends far beyond the period of active treatment with the drug.

## Additional files

10.1186/s12933-016-0332-6 Baseline Clinical and Laboratory Characteristics of the study cohort by original study allocation.
10.1186/s12933-016-0332-6 Baseline Clinical and Laboratory Characteristics of the study cohort by triglyceride level ≥ and < 200 mg/dl.

## Abbreviations

AP: angina pectoris
BMI: body mass index
CHD: coronary heart disease
DM: diabetes mellitus
HDL-C: high-density lipoprotein cholesterol
LDL-C: low-density lipoprotein cholesterol
LLD: lipid lowering drugs
MI: myocardial infarction
TG: triglycerides
BIP: Bezafibrate Infarction Prevention
