# Supplementary material for: Bezafibrate for the treatment of dyslipidemia in patients with coronary artery disease: 20-year mortality follow-up of the BIP randomized control trial
Source: Cardiovasc Diabetol. 2016 Jan 22;15:11. doi: 10.1186/s12933-016-0332-6 (PMC4722704; doi:10.1186/s12933-016-0332-6)
Supplement: Supplementary file 1 — 10.1186/s12933-016-0332-6 Baseline Clinical and Laboratory Characteristics of the study cohort by original study allocation. [file 12933_2016_332_MOESM1_ESM.docx]

Supplemental data – online only

Additional file Table A - supplemental. Baseline Clinical and Laboratory Characteristics of the study cohort by original study allocation ^&^

|  | **Bezafibrate Group** (n = 1,539) | **Placebo Group**  (n = 1,532) |
| --- | --- | --- |
| **Clinical characteristics** |  |  |
| Age, yrs | 61 ± 7 | 61 ± 7 |
| Male | 665 (90) | 619 (92) |
| Hypertension | 220 (30) | 221 (33) |
| DM | 73 (10) | 74 (11) |
| BMI, kg/m^2^ | 26.7 ± 3.4 | 26.7 ± 3.3 |
| NYHA functional class ≥2 | 201 (28) | 168 (26) |
| AP functional class ≥2 | 219 (30) | 184 (27) |
| Prior MI | 586 (80) | 541 (81) |
| Prior angina | 438 (60) | 400 (60) |
| COPD | 25 (3) | 29 (4) |
| Triglycerides ≥ 200 mg/dl | 270 (15) | 256 (15) |
| **Medical therapy** |  |  |
| Anti-platelets | 1085 (70) | 1060 (69) |
| Beta-blockers | 580 (38) | 604 (39) |
| Nitrates | 789 (51) | 779 (51) |
| Ca^2+^-blockers | 773 (50) | 798 (52) |
| ACE inhibitors | 185 (13) | 196 (14) |
| Diuretics | 210 (12) | 222 (13) |
| Non-study LLD | 810 (53) | 868 (57)** |
| **Laboratory values** |  |  |
| Total cholesterol | 209 ± 17 | 209 ± 17 |
| HDL-C | 35 ± 6 | 35 ± 5 |
| LDL-C | 146 ± 16 | 146 ± 16 |
| Triglycerides | 134 ± 44 | 136 ± 43 |
| Fibrinogen | 349 ± 72 | 349 ± 72 |

Values are presented as n (%) or mean ± SD.

^&^P-value > 0.1 for all comparisons with the exception of non-study LLD use during follow-up period p=0.02

^**^ p value < 0.01

AP = angina pectoris; ACE = angiotensin-converting enzyme; BMI = body mass index; COPD = chronic obstructive pulmonary disease; DM = diabetes mellitus; HDL-C = high-density lipoprotein cholesterol; LDL-C = low-density lipoprotein cholesterol; LLD = lipid-lowering drug; MI = myocardial infarction; NYHA = New York Heart Association.
