# Supplementary material for: Bezafibrate for the treatment of dyslipidemia in patients with coronary artery disease: 20-year mortality follow-up of the BIP randomized control trial
Source: Cardiovasc Diabetol. 2016 Jan 22;15:11. doi: 10.1186/s12933-016-0332-6 (PMC4722704; doi:10.1186/s12933-016-0332-6)
Supplement: Supplementary file 2 — 10.1186/s12933-016-0332-6 Baseline Clinical and Laboratory Characteristics of the study cohort by triglyceride level ≥ and < 200 mg/dl. [file 12933_2016_332_MOESM2_ESM.docx]

Additional Table B - Baseline Clinical and Laboratory Characteristics of the study cohort by triglyceride level ≥ and < 200 mg/dl.

| **Clinical characteristics** | **TG < 200 mg/dL** (n = 2,613) | **TG ≥ 200 mg/dL** (n = 458) | **P value** |  |
| --- | --- | --- | --- | --- |
| Age, years | 60 ± 7 | 58 ± 7 | < 0.001 |  |
| Male | 2,383 (91) | 409 (90) | 0.27 |  |
| Hypertension | 811 (32) | 158 (34) | 0.20 |  |
| DM | 256 (10) | 49 (11) | 0.73 |  |
| BMI, kg/m^2^ | 26.5 ± 3 | 27.9 ± 3 | < 0.001 |  |
| NYHA functional class ≥ 2 | 627 (24) | 124 (27) | 0.39 |  |
| CCS Angina score ≥ 2 | 653 (25) | 125 (27) | 0.33 |  |
| Family History of CAD | 993 (38) | 183 (40) | 0.38 |  |
| Active Smoker | 285 (11) | 77 (17) | < 0.001 |  |
| Prior MI | 2038 (78) | 344 (75) | 0.66 |  |
| COPD | 74 (3) | 12 (2) | 0.28 |  |
| **Medical therapy** |  |  |  |  |
| Anti-platelets | 1821 (70) | 312 (68) | 0.16 |  |
| Beta-blockers | 967 (37) | 210 (46) | 0.01 |  |
| Nitrates | 1316 (51) | 241 (53) | 0.86 |  |
| Ca^2+^-blockers | 1338 (51) | 223 (50) | 0.47 |  |
| ACE inhibitors | 319 (12) | 57 (12) | 0.93 |  |
| Diuretics | 357 (14) | 63 (14) | 0.84 |  |
| Oral diabetic treatment | 131 (5) | 27 (6) | 0.30 |  |
| Use of non-study LLD* | 1378 (54) | 256 (56) | 0.38 |  |
| **Laboratory values** |  |  |  |  |
| Total cholesterol mg/dl | 212 ± 17 | 215 ± 17 | < 0.001 |  |
| HDL-C mg/dl | 35 ± 6 | 31 ± 5 | < 0.001 |  |
| LDL-C mg/dl | 149 ± 16 | 144 ± 17 | < 0.001 |  |
| Triglycerides mg/dl | 129 ± 35 | 237 ± 34 | < 0.001 |  |
| Glucose mg/dl | 101 ± 22 | 106 ± 26 | < 0.001 |  |
| Creatinine mg/dl | 1.14 ± 0.1 | 1.13 ± 0.2 | 0.62 |  |
| Fibrinogen | 348 ±73 | 357±74 | 0.01 |  |

Values are presented as n (%) or mean ± SD.

AP = angina pectoris; ACE = angiotensin-converting enzyme; BMI = body mass index; CCS- Canadian Cardiovascular Society; COPD = chronic obstructive pulmonary disease; DM = diabetes mellitus; HDL-C = high-density lipoprotein cholesterol; LDL-C = low-density lipoprotein cholesterol; LLD = lipid-lowering drug; MI = myocardial infarction; NYHA = New York Heart Association. * Use on non-study LLD any time during the study or extended follow-up period (median 7.9 years from study initiation).
